# Secrecy strategies: Global patterns in elites’ quest for confidentiality in offshore finance

**DOI:** 10.1371/journal.pone.0326228

**Published:** 2025-07-16

**Authors:** Ho-Chun Herbert Chang, Brooke Harrington, Daniel Rockmore

**Affiliations:** 1 Program in Quantitative Social Science, Dartmouth College, Hanover, New Hampshire, United States of America; 2 Department of Sociology, Dartmouth College, Hanover, New Hampshire, United States of America; 3 Department of Mathematics, Dartmouth College, Hanover, New Hampshire, United States of America; Alexandru Ioan Cuza University: Universitatea Alexandru Ioan Cuza din Iasi, ROMANIA

## Abstract

Scholars and policy-makers know a lot about the ways offshore financial centers compete with one another to offer secrecy to elites, but still know too little about how and why elites take up these offerings to conceal their assets and identities offshore. This paper fills the gap in knowledge by examining the distinct patterns for achieving offshore secrecy among elites in 65 countries. We take a cross-national comparative perspective, showing that the patterns are contingent in part on political conditions in the elites’ home countries. Using data from two publicly available sources— the Offshore Leaks Database and the World Justice Project Rule of Law Index—we advance knowledge for scholars and policy-makers with three main results. First, we find that elites from corrupt countries are more likely to spread their assets across multiple offshore financial centers: they diversify across the system, instead of putting all their eggs in one basket. Second, countries where the risk of government confiscation of private assets is high—either due to lack of civil rights or very effective law enforcement—elites make heavy use of identity-concealing offshore strategies such as bearer instruments and nominees to shield their names from discovery in public records. Third, elites from countries where both corruption and confiscation pose significant risks make extensive use of blacklisted offshore financial centers, despite the reputational and practical risks that entails for them. All three patterns achieve secrecy, but through different means; our findings have implications for public policy, as well as for models of inequality, elites, and financial crime.

## 1 Introduction

Elites’ use of the offshore financial system appears to be highly patterned, shaped by the legacies of past political, economic and social structures [1]. These legacies include histories of colonialism and communism, as well as corruption and the rule of law [2,3]. Recent research shows how valuable “studies of” such patterns can be in analyzing and predicting financial crime: for example, Chang *et al*. (2023) pointed to previously unknown vulnerabilities in the offshore networks of Russian elites sanctioned following the 2022 invasion of Ukraine, with significant implications for scholarly models of inequality as well as for public policy [4]. In particular, the findings underscored the need for additional scrutiny of professional intermediaries—wealth managers—in creating the patterns researchers observe [5,6].

Yet despite these advances, understanding of patterns in the transnational networks of offshore finance has been tightly limited by the secrecy surrounding the system and the scarcity of available data. At the same time, secrecy is the most important product of the offshore system and the phenomenon most urgently in need of analysis [7]. Better understanding of the ways secrecy is organized and patterned would contribute not only to better policies but to better theories of stratification, elites and financial crime. This paper extends the emergent line of inquiry on patterns in elites’ use of offshore finance by focusing on their secrecy strategies.

Our analysis examines the institutional conditions that motivate elites’ use of the offshore system, with emphasis on the roles of corruption and rule of law in their home countries. To conduct this investigation, we link two publicly-available datasets. For data on patterns in elites’ use of offshore finance, we draw on the Offshore Leaks database from the International Consortium of Investigative Journalists: 6.9 terabytes of data and tens of millions of records derived from the 2016 Panama Papers, 2017 Paradise Papers and 2021 Pandora Papers, among other sources. Our research is the first to analyze this unique data resource in full. For information on the institutional conditions in the elites’ home countries, we use the World Justice Project Rule of Law Index, which compiles data for 142 countries on their levels of civil and criminal justice, corruption, government transparency and regulatory enforcement [8].

Our analysis yields three main results. First, we find that when elites come from countries high in institutional corruption, they are more likely to spread their assets across multiple offshore financial centers: they diversify, in what might be called a “don’t put all your eggs in one basket” strategy. Second, we find that for countries where the risk of government confiscation of assets is high—either due to lack of civil rights or due to very effective law enforcement—elites make heavy use of secrecy strategies that conceal their identities as asset owners; this includes the use of anonymous bearer instruments, as well as the employment of nominees to shield elites’ names from discovery in public records. Third, we find that elites from both types of countries make frequent use of blacklisted offshore financial centers. Blacklisted jurisdictions are countries sanctioned by international bodies like the OECD or European Union for their high levels of secrecy—refusing to share information—and for facilitating tax avoidance and evasion. Using them to hold assets entails considerable reputational risks and increased transaction costs for elites [9,10]. Overall, our most counter-intuitive finding is that use of offshore finance by elites can be driven not only by poor governance in their home countries—like corruption and lack of civil liberties—but by good governance conditions, as well. Thus, our 65-country analysis offers useful implications for public policy, as well as scholarly models of inequality, elites and the geography of finance.

## 2 Methods

### 2.1 Data sources

Our analysis draws on the best available data on elites’ use of offshore, in the form of the Offshore Leaks dataset, made public by the International Consortium of Investigative Journalists. This is the largest and most globally comprehensive database on offshore finance, containing records drawn from sources including: the Offshore Leaks (2013) comprised approximately 2.5 million files totaling about 260 gigabytes of data; the 2016 Panama Papers, comprising 2.6 terabytes of data and 11.5 million documents from the Panamanian law firm Mossack Fonseca; the 2017 Paradise Papers, comprising 1.4 terabytes of data and 13.4 million documents from the Bermuda law firm Appleby and the Hong Kong corporate service firm Asiaciti Trust; and the Pandora Papers leak of 2021, comprising 2.9 terabytes of data and 11.9 million documents from 14 different organizations providing offshore financial services.

All of these data sources reveal what are otherwise highly secretive personal financial affairs of ultra-high-net-worth individuals from around the world. Since it is very costly to use the offshore financial system [5], the individuals named in the Offshore Leaks database—which include a wide range of heads of state, celebrities and corporate leaders—all have two things in common: they are extremely wealthy and have something to hide. This group includes, but is not limited to, most of the approximately 3,000 billionaires in the world. While some may turn to the offshore financial system to avoid taxes, their more general motive is to conceal their ownership of certain assets—in particular, how the assets were acquired and how they have been (or will be) spent. In addition, elites from some countries have well-founded reasons to fear that their assets might make them targets of kidnapping or extortion, or even political reprisals from their own governments [2,5]. Whatever their motive, elites purchase offshore secrecy in order to break the chain of association in law and public records linking them to their wealth [7].

To understand better the conditions motivating elites to use the offshore financial system, we turn to the World Justice Project Rule of Law Index. The Index is used as the basis for other metrics created by the World Bank, Transparency International and private sector organizations like financial ratings agencies. However, our work is the first to connect offshore finance data with Rule of Law indicators. These indicators are included in the Supporting information (S1 Table).

The Rule of Law Index is an integrative statistic built from over 400 variables, measuring the ability of a country to deliver on the “rule of law” construct. The WJP defines the construct as follows: “a durable system of laws, institutions, norms, and community commitment that delivers four universal principles: accountability, just law, open government, and accessible and impartial justice.” The Index is built from two data sources: general population surveys designed by the WJP with at least 1,000 respondents; and qualified respondent questionnaires completed by in-country experts on civil, commercial, criminal, and labor law. It has been computed annually since 2008 and now covers 142 countries and jurisdictions. Our analysis uses the Index to construct regression models assessing how rule of law (or lack of it) shapes the offshore secrecy strategies of elites. Assuming the WJP Index rankings remain somewhat stable over time, our analysis of elites’ offshore activities over the past two decades will yield a snapshot of the correlations between their secrecy strategies and the rule of law in their home countries. For this purpose, we chose the 2015 and 2016 WJP Rule of Law Index, as the majority of the events in the ICIJ dataset occur after those years. The dataset is composed of the Offshore Leaks (2013), the Panama Papers (2016), the Paradise Papers (2017), FinCEN Files (2020), and Pandora Papers (2021).

Lastly, to compute inclusion in “blacklist” regions, we use the powerset of all current and previously sanctioned jurisdictions from the European Union (EU 2017 and 2020), Financial Action Task Force (FAFT 2016, 2017, 2020), along with the original OECD blacklist from 2000. The full list is included in Supporting information S2 Table. The purpose of using a powerset is because offshore secrecy has been an active area of policy reform, such as the implementation of the Common Reporting Standard [11]. Checks on all powersets increase confidence in the robustness of our metrics.

Blacklisting a country means sanctioning it by cutting it off from certain types of financial and legal transactions for one or both of the following reasons: 1) harmful tax practices (meaning low or nil tax regimes that undercut the revenues gathered by other countries); 2) excessive secrecy, meaning a pattern of refusing to share information about assets other countries’ nationals (and firms) might have in the blacklisted jurisdiction. Individual countries can blacklist other countries, but the most compelling sanctions are leveled by multi-national organizations, such as the 38-member Organization for Economic Cooperation and Development (OECD), the 27-nation European Union or the 39-member Financial Action Task Force. Those organizations sanction non-cooperative offshore jurisdictions as part of their mission. For example, blacklisting is described as “part of the EU’s work to fight tax evasion and avoidance” [12]. The FATF pursues blacklisting as part of its foundational remit from the G7 to “develop policies to combat money laundering” [13].

When the EU blacklists a jurisdiction, that means no EU funding can be channeled through that country, and any financial schemes routed through the sanctioned country by an EU firm or national can be subjected to additional reporting requirements, monitoring and tax withholding rates; any transactions in a blacklisted jurisdiction are thus subject to significantly higher transaction costs, as well as reputational stigma [14]. Blacklisting efforts by other organizations and countries are similarly designed to reduce financial flows to listed jurisdictions by significantly raising the costs—in money, time and reputation—of holding assets there. It is therefore noteworthy and surprising when firms and elites, who are ordinarily very protective of their reputations [15], are willing to engage with blacklisted jurisdictions at all. Even when blacklisted countries can get themselves removed from sanctions lists, the lingering reputational costs of doing business there can remain substantial [9].

As must also be acknowledged, scholars have documented that blacklists are political documents lacking objective criteria, and therefore susceptible to political manipulation [16]. For instance, many of the listed jurisdictions are small post-colonial states with little political power to advocate for themselves on the world stage [17–19]. To counteract potential bias due to using one well-known blacklist in our analysis, we conducted robustness checks using a powerset of multiple lists, then included robustness tests using the Basel Index and the Financial Secrecy Index (FSI) created by the Tax Justice Network [17]. Those results align with our blacklist findings and corroborate the robustness of our approach.

**Ethics statement.** This study did not require Institutional Review Board (IRB) approval because it relied exclusively on publicly available and did not involve human participants as defined by the Declaration of Helsinki.

### 2.2 Key metrics

We analyze three key metrics of secrecy: 1) diversification in the use of offshore financial centers; 2) use of identity concealment strategies; and 3) use of blacklisted offshore jurisdictions, including diversification in the use of blacklisted jurisdictions. To operationalize diversity, we use the Shannon entropy measure, which was developed in the biological sciences [20,21] but now commonly used in social science and social networks [22–26]. Entropy is the expectation of the log probabilities over all possible offshore locations. This is given in Eq 1:

H=−∑p(x)logp(x) (1)

where each *p*(*x*) denotes the proportion of clients that have assets in a given offshore location *x*, over all clients of one country. Since *p*(*x*) represents a proportion, the sum of all *p*(*x*) is 1. We measure the use of offshore diversification strategies to achieve offshore secrecy by computing for every country of origin (*x*) for elites named in the Offshore Leaks database. This yields Eq 2:

$$H_{Offshore}(x)=-\sum_{p\in OFF}p\log p$$ (2)

where *OFF* is the proportion of entities—firms, trusts and foundations—that elites from country *x* allocate to each offshore jurisdiction. An example with BRICs countries is given in Supporting information S1 Fig.

To operationalize the second main secrecy strategy, identity concealment, we combine two factors: the use of bearer shares and bonds, as well as of nominee shareholders and directors. Bearer shares and bonds are like regular stocks and bonds, except that they are not made out in the name of any particular owner; like a check made out to “cash” instead of to an individual, whoever holds the piece of paper is the legal owner of the asset [27]. This means that anyone wishing to conceal their ownership of assets in an offshore corporation can legally and truthfully deny any association with those assets, as long as they are not holding the bearer certificates at the moment [28]. This is useful in judicial proceedings where a government or creditor might wish to tax or confiscate an asset belonging to a high-net-worth individual.

An even more common way of achieving the same result is through the use of nominee shareholders or directors. Nominees are people who “rent” their names to help elites avoid public disclosure requirements that would otherwise reveal the true names of the owners and managers of an offshore company [27]. As a practical matter, this involves inserting the equivalent of the name “John Doe” in all public-facing documents where the names of the true shareholders and directors of a firm would otherwise be shown. The nominees themselves have no powers of ownership or control, serving only to preserve the anonymity of the elites who purchase the use of their names.

Thus, we operationalize identity concealment strategies as follows. First, for every client in the Offshore Leaks database, we count the number of nominees and bearer instruments their wealth manager employs, divided by the wealth managers’ total number of clients. Then, for every client from country *x*, we take the average; this captures the propensity of elites from each country to choose wealth managers whose offshore finance strategies focus on identity concealment. This results in Eq 3:

$$IC(x)=\frac{1}{\left|x\right|}\frac{\sum Anon(j)\forall j\in Man(i)\forall i\in x}{\left|Man(i)\forall i\in x\right|}$$ (3)

where *i* is a client and *x* is the set of clients from a specific country. *Man*(*i*) represents the total set of clients a wealth manager of *i* works with globally, while *Anon*(*j*) represents the number of that wealth manager’s clients whose offshore financial arrangements involve the use of nominees and/or bearer instruments.

Finally, we analyze in two ways elites’ use of blacklisted jurisdictions as a means to conceal their ownership of offshore assets. First, we measure the percentage of each individual’s total offshore structures (the number of firms, trusts and foundations associated with them) that are based in blacklisted jurisdictions. Second, we measure jurisdictional diversification in elites’ use of blacklisted offshore centers.

Operationally, we measure the use of this strategy as the percentage of blacklisted jurisdictions (based on our aforementioned powerset of the FAFT, OECD, and EU) used by elites from a given country. This is aggregated at the country level, as shown in Eq 4 below:

$$\%BL(x)=\frac{\text{\# of blacklist for country }x}{\text{Total \# of offshore locations for country }x}$$ (4)

We calculate this for every country *x* where elites in our data originate.

As a control, we also weight these jurisdictions with scores from the Basel Anti-Money Laundering (AML) Index, which is considered a gold standard for assessing the risk of money laundering and terrorist financing [29]. We use two specific metrics. First, the AML and CFT (Combating the Financing of Terrorism, a sub-index of Basel) framework provide a snapshot of the laws around anti-money laundering, and hence an expert measure of secrecy. Second, financial transparency is the extent to which companies in a jurisdiction provide clear and accessible information. The weighted score ML-risk is then described in Eq 5:

$$\overset{\sim }{\mathrm{ML}}(x)=\sum p(y)ML(y)$$ (5)

where *ML*(*y*) denotes the ML score based on the Basel Index for country *y*, *y* denotes an offshore center that an elite from country *x* utilizes, and $\overset{\sim }{\mathrm{ML}}(x)$ the aggregate score. A similar computation is carried out for financial transparency ($\overset{\sim }{\mathrm{FT}}(x)$). These results are shared in S4 Fig of the Supporting information. We also use the Financial Secrecy Index (FSI)—which is the second component of the Basel Index and known for its objectivity—to compute a weighted score in a similar way. Like the WJP indices, we use the 2015 and 2016 Basel Index and Financial Secrecy Index (FSI), since they precede the majority of these leaks.

Finally, we use the entropy calculation to measure the extent of diversification in elites’ use of blacklisted jurisdictions to preserve secrecy around their ownership of assets. For every country *x* where the elites in our dataset originate, we compute:

$$H_{Blacklist}(x)=-\sum_{p\in BL}p\log p$$ (6)

where BL is the set of percentages for each blacklisted destination according to our composite blacklist.

After generating the country-level ICIJ dataset—based on the origin countries of elites—we then merged that information with the WJP Rule of Law Index from 2015 (the mean year of the leaks). For countries that were as yet unrated in the Rule of Law Index by that year, we chose the closest subsequent year. This yielded a total of 65 countries for our analysis. The values for the 44 WJP indicators and 8 macro-categories were normalized between 0 and 1 for the regression.

## 3 Results

In this section, we first offer some illustrations of the varying patterns in use of offshore financial centers and strategies among elites from the 65 countries in our analysis. This analysis draws on the metrics we developed to measure diversification in the use of offshore financial centers, as well as the use of identity-concealment strategies (like bearer instruments and nominees), and the decision to place assets in blacklisted jurisdictions. Next, we examine some possible motivations driving these strategies, drawing from the World Justice Project Index measures of institutional governance conditions in elites’ home countries.

**Fig 1:**
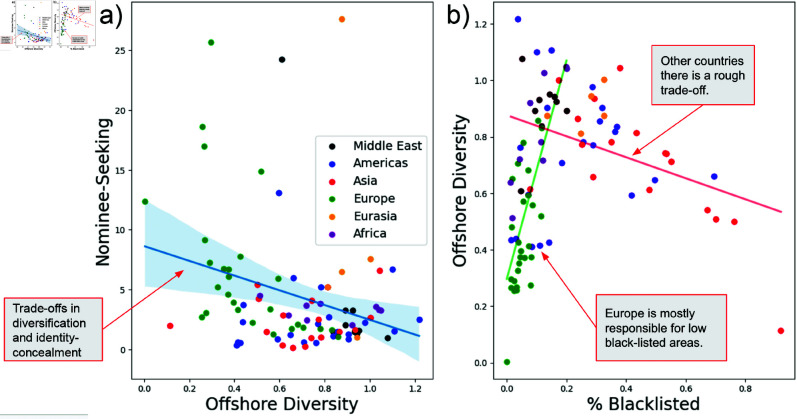
Trade-offs between offshore strategies. Fig 1a) shows the trade-off between diversification and the use of identity-concealment strategies via nominees and bearer instruments. Fig 1b) shows the trade-off between elites’ allocation of their assets to blacklisted jurisdictions and their use of diversification strategies.

We find significant diversity in patterns of offshore use based on elites’ home countries. Fig 1 offers an initial look at these findings. Fig 1a) shows the relationship between strategies of diversification in the use of offshore financial centers (x-axis) and the use of identity-concealment strategies via nominees and bearer instruments (y-axis). The downward slope of this graph indicates a trade-off: as elites diversify in the number of offshore financial centers used to hold their assets, they become less likely to use identity concealment strategies.

Fig 1b) examines the relationship between elites’ allocation of their assets to blacklisted jurisdictions (x-axis) and their use of offshore diversification strategies (y-axis). The data here show that elites from countries in Europe and the Middle East make little use of blacklisted offshore financial centers, except when their overall offshore diversification increases. In contrast, elites from the rest of the world (Americas, Asia, Eurasia, and Africa) show the opposite pattern: the more use they make of blacklisted jurisdictions, the lower their overall offshore diversification. This means elites from those four regions tend to concentrate their assets in just a small number of blacklisted offshore centers. Thus, the entropy measure of diversification in use of blacklisted jurisdictions is very low for China (0.13), Russia (0.23), and Brazil (0.14) compared to Great Britain (0.57) and the US (0.52).

**Fig 2:**
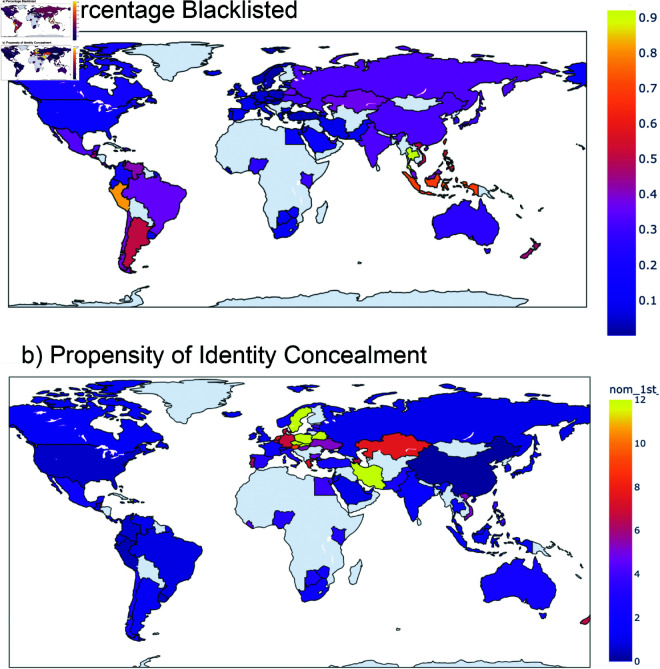
Geolocation of strategies. Percentage of blacklisted jurisdictions used (a) and propensity of identity concealment (b). These maps were created using Plotly’s Choropleth.

Fig 2 offers another view of elites’ patterns in the use of offshore finance by presenting a “heat map” comparing the intensity in uptake of two strategies: Fig 2a) shows the percentage of offshore assets allocated to blacklisted jurisdictions; Fig 2b) shows the mean frequency in the use of nominees and bearer instruments to conceal elites’ identities. Countries not represented in the data are in gray. These maps were created using Plotly’s Choropleth.

We find a surprisingly high uptake in the use of blacklisted jurisdictions, given the additional costs imposed by that strategy. As Fig 2a) shows, elites from Peru, Thailand, Indonesia, and Malaysia allocate 70-90% of their offshore assets to blacklisted jurisdictions; elites from Mexico, Brazil, Russia, India and China allocate about 30% of their offshore assets to blacklisted offshore centers. Elites from Europe and Middle East rarely place their assets in those jurisdictions. Supporting information S1 offers further detail on the allocation of assets to blacklisted countries, showing the dominance of the British Virgin Islands as an offshore destination.

The map of identity concealment strategies (Fig 2b) is equally surprising, in that we find the highest uptake among an unlikely assortment of countries: Sweden, Iran, Poland, Belarus, Kazakhstan, and Germany. This requires further analysis, leading us to the regressions in the next section, informed by the World Justice Project Rule of Law Index.

### 3.1 Regression results

To explain why elites from different countries exhibit such distinctive and sometimes surprising variations in their offshore secrecy strategies, we turn to regression models linking indices from the World Justice Project to four outcomes: identity concealment; use of blacklisted jurisdictions; general diversification in the use of offshore financial centers; and diversification in the use of blacklisted centers. Overall, we find that identity concealment strategies are most widely used by elites from corrupt countries with high levels of civil justice, while use of blacklisted jurisdictions is most common in the opposite conditions: when elites are from countries with low levels of corruption and civil justice. In terms of diversification, elites from countries with low levels of fundamental rights and low levels of order and security spread their assets across a wide range of offshore financial centers; in contrast, elites from countries that are seemingly well-run (with high levels of regulatory enforcement and fundamental rights) are most likely to use a diverse range of blacklisted offshore centers.

Our regression model is built on CatBoost (Categorical Boosting Trees), a variant of gradient-boosting trees [30]. Gradient-boosting trees are ensembles of decision trees, each a weak learner that models the underlying system. As the sample size is smaller, we restrict the maximum tree depth to three, which allows at most only three-way interactions between variables. This reduces the likelihood of overfitting. Although machine learning techniques like gradient-boosting can capture nonlinear interactions better and hence yield higher accuracies than base statistical methods, their “black box” nature has limited their interpretability and thus application to social scientific questions. SHAP explainers have emerged as a tool to improve machine learning explainability; they quantify the amount that covariates contribute to a model. SHAP explainers are based on Shapley Values in game theory [31], which evaluate the contribution of an individual in a coalition game. In studies like ours, SHAP values evaluate power sets of features and their contributions to minimizing error in the model [32].

**Fig 3:**
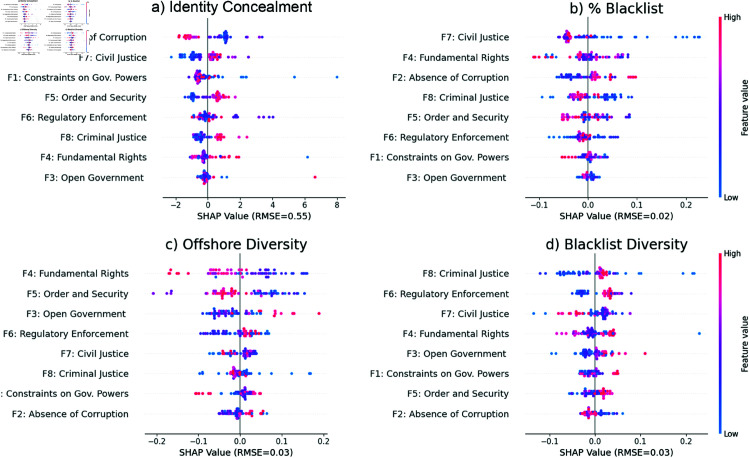
SHAP values for offshore strategy metrics. SHAP values for a) identity concealment strategies used, b) percentage of blacklisted jurisdictions used, c) general diversification in use of offshore financial centers, and d) diversification in use of blacklisted offshore financial centers.

Fig 3 shows the shows regression results based on the WJP macro-categories and RMSE values. Features are listed top to bottom by their importance; the top three indicators have the greatest share of model contribution (similar to explained variance). Each dot represents a SHAP value for each sample (one country) on one of the eight dimensions. The idea is to evaluate factors that have clear positive or negative correlations with these outcome variables. Color represents the feature value: clear red on the right and blue on the left indicate a positive correlation. Full ${\text{r}}^{2}$ for model fits are included in Supporting information S3 Fig and robustness checks with the Basel Index and FSI in S4 Fig.

Civil justice in elites’ home countries is one of the most important “predictors” in their use of identity concealment strategies and blacklisted jurisdictions. In the WJP Index, a lack of civil justice means that legal remedies for everyday problems—through courts or law enforcement—are inaccessible to most (see Table S1). This inaccessibility may be due to problems of cost, political bias, discrimination, unreasonable delays, or other factors. In such conditions, elites seem most concerned with disguising their connections to their own wealth, perhaps due to fears of confiscation [2].

Our second main finding is that as corruption increases in elites’ countries, they diversify in their allocation of assets to offshore jurisdictions overall, as well as to blacklisted jurisdictions. The WJP Index defines corruption to mean government officials’ use of public office for private gain—for example, through bribery and kickbacks (see Table S1). Under those conditions, elites seem less concerned with being identified with their wealth than with the possibility of it all being discovered at once. Therefore, they spread their wealth over numerous offshore centers, so that an incursion on one asset does not affect the others. This confetti-like scattering is a common secrecy strategy for terrorist cells and financial fraudsters [33].

Our third main finding is that as order and security increases in their home countries, elites make more use of blacklisted jurisdictions and diversify their use of offshore financial centers in general. The WJP Index defines order and security as effective control of crime and civil conflict. In other words, as a society becomes better at ensuring the security of persons and property, the more its wealthiest members turn to the offshore financial system. This is consistent with economic research showing that elites from Scandinavia and elsewhere in northern Europe make surprisingly common use of offshore to escape high tax rates and ruthlessly efficient regulatory enforcement [34].

This suggests a counterintuitive result: use of offshore finance is driven not only by negative political conditions such as corruption and lack of civil justice, but by positive conditions, such as regulatory enforcement and civil justice. These positive conditions have received very little attention, but are consistent with earlier sociological research on paradoxes in countries’ development of informal economic sectors: parts of the economy that are unregulated, untaxed, and sometimes illegal, ranging from under the table payments to nannies and plumbers, to organized drug rings. Portes (1994) and subsequent researchers showed that the size of countries’ informal economies could be represented as a U-shaped curve, because the informal sector was largest in both the worst- and best-governed nations [35]. In the latter case, the motivation to use the informal sector for transactions was to avoid “too much” order and security in the form of taxes and regulations; similar motivations may drive elites in those countries to use the offshore financial system.

**Fig 4:**
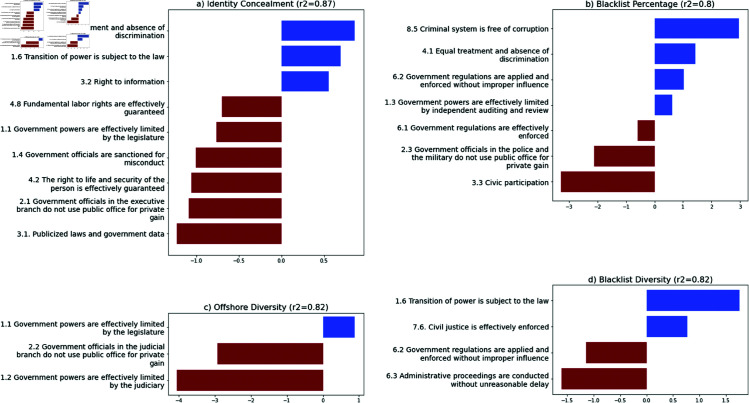
Statistically significant indicators for the four offshore metrics. (a) identity concealment, (b) blacklist percentage, (c) offshore diversity, and (d) blacklisted diversity.

Next, we look deeper into the factors driving each of the four strategies by considering the sub-categories within the Rule of Law Index. Each of the horizontal bar graphs in Fig 4 represents the coefficient for statistically significant indicators, and can be interpreted as both the magnitude and direction of effect.

Fig 4a) shows that countries where elites make most use of identity concealment strategies are those where citizens have strong legal rights to information, but at the same time experience very low levels of transparency in terms of government data and laws. While this may seem contradictory, it may reflect the same kind of bimodal relationship we find with both the “civil justice” and “corruption” variables. That is, elites from two very different kinds of countries may both turn to identity concealment strategies for opposing reasons. Nations where information is publicly available, accessible, and ready for audit—indicating good governance and transparency—may also impose high taxes and strict regulations, motivating wealthy citizens to seek identity concealment offshore. Denmark and Austria are good examples of this combination of good governance and high usage of identity concealment strategies.

In contrast, countries characterized by poor governance measures, such as obscuring their citizens’ legal rights and data, produce elites who use nominees and bearers to disguise their ownership of offshore assets. One example is Iran, which ranks low on the WJP Index in terms of legal transparency (3.1) and the second-lowest in guaranteeing citizens’ right to life and security (4.2). Unsurprising then that Iranian elites make frequent use of identity concealment strategies like nominees to disguise their ownership of offshore assets. This again points to the thesis that both positive and negative institutional conditions drive the use of offshore finance.

Fig 4(b) suggests a similar bimodal pattern, in that the use of blacklisted jurisdictions is catalyzed both by good governance and bad. Countries where civic participation is low due to fear of retribution from the government produce elites who use blacklisted locations to disguise their ownership of offshore assets. This is likely the case for China, which has the lowest level of civic participation in the WJP Index, and where protestors in civil demonstrations have shown particular care in disguising their identities [36]. In other words, low levels of civic participation have the biggest impact on elites’ allocation of their assets to blacklisted countries.

But several measures of good government—such as lack of corruption in the criminal justice system, and properly enforced government regulation—have an equal or greater influence in driving the use of blacklisted offshore centers. Some countries, such as Singapore, embody the full paradox in that their governments are fair and largely free of corruption, but civic participation nonetheless remains low and there are strong social and political motivations to remain anonymous about wealth ownership [37,38].

Fig 4(c) offers a somewhat more one-sided story, showing that diversification in the use of offshore financial centers is driven mostly by government dysfunction—particularly in countries where the judiciary has little power to constrain other branches of government. Per the WJP Index data, countries such as Ukraine and Vietnam fall into this category; while their laws may be well-publicized and protect freedom of religion, there are few judicial checks on government power to confiscate assets or exact retribution against political enemies. This appears to drive a strategy in which elites scatter their assets across a variety of offshore financial centers.

Lastly, Fig 4(d) offers a story about corruption and bad governance. Elites use of a diverse range of blacklisted offshore jurisdictions is highest in countries where government is not subject to effective oversight, and in which application of the law is unfair and proceedings are unreasonably slow. Examples include Liberia, Belize, and to a lesser extent, South Africa.

## 4 Discussion

This study contributes novel insights to the emerging line of research on the patterned uses of offshore finance by high-net-worth individuals. We first document new patterns in offshore activity among elites from 65 countries, using data from the Offshore Leaks Database of the ICIJ. Our analysis identifies three secrecy strategies employed, alone or in combination, by the elites who appear in the ICIJ’s six datasets of offshore financial leaks. Because these data are definitionally incomplete, we cannot know for certain if these strategies we have identified are in fact the dominant ones in use by elites across the global system of offshore finance. Qualitative research suggests that they are [5], but it remains for future leaks and future research to establish a clearer understanding of the range and frequency of strategies employed by elite clients of the offshore financial system.

The concepts of jurisdictional diversification and identity concealment as distinct secrecy strategies are new contributions to the scholarly debates; though the two strategies may be combined, our analysis indicates that they are more usually used separately. Similarly, we find a surprisingly high allocation of assets to blacklisted jurisdictions by elites from countries that would otherwise seem to have little in common. This finding suggests a need for further research on the global linkages among elites across nationalities and cultures [7].

Regression analysis against covariates from the World Justice Project Index allow us to examine the drivers of these patterns in offshore secrecy strategies, linking them to institutional conditions in elites’ home countries. In general, we find that elites’ offshore diversification strategies are driven by corruption in their home countries’ governments. In contrast, identity-concealment strategies are preferred by elites from countries where governments know “too much” about their citizens—either because the governments are autocracies or because the governments are rigorously fair and strict in their applications of the law. This suggests that patterns in the use of offshore finance stem not only from negative political conditions in elites’ home countries, but positive ones normally associated with good governance.

Our study has a few limitations. Due to the nature of these leaks, there may be inherent exposure biases due to geographical correlation. This is a shared concern for studies focused on secrecy phenomena— data are often available as incomplete snapshots of the overall behavior. For the offshore financial system, the ICIJ datasets provide the most comprehensive view available of offshore financial activity. Future research that estimates the possible geographical variance can help qualify our analysis.

Another limitation is the correlational nature of our analysis. As with most social systems, these systems are feedback loops and elites are not a monolith in terms of their motivations for using the offshore system. Previous qualitative research on offshore finance indicates that some elites use the system to avoid the threats of confiscation from authoritarian regimes in their home countries, while other elites simply wish to avoid scrutiny [5]. This could be for a variety of reasons, from fear of personal embarrassment (e.g., the revelation of an extramarital affair or of religious non-compliance), as well as the desire to conceal illegal activities, such as tax evasion. While our goal was to articulate a typology of strategies, further research investigating the sequential and temporal dimension would help elucidate cause and effect more precisely.

These results will be of interest for research on elites, inequality, and the geography of finance—particularly our findings on counter-intuitive patterns in the use of offshore by elites from countries characterized by good governance. Many such countries, in their efforts to stem rising inequality through strict taxation and regulation, may also be motivating some of their wealthiest citizens to develop offshore secrecy strategies. This would be consistent with recent economic research on Scandinavians’ use of offshore [34], as well as sociological research on the paradoxes of the information economy [35]. For policy-makers, our findings may be useful in predicting problematic offshore activity by elites based on changing governance conditions in their home countries. Simulations based on our model could generate the kind of information that until now has mainly been accessible only through leaks like the Panama Papers. We offer our results as a platform for further investigation.

## Supporting information

S1 Fig Distribution of offshore assets by client country. BRICS countries rely dominantly on the British Virgin Islands (VGB). S1 Fig shows the exact distribution of where clients hold their assets in blacklisted regions. Brazil, Russia, India, and China rely almost exclusively on the British Virgin Islands. In contrast, South African clients tend to rely on the Jersey islands. We also quantify the diversity using Shannon Entropy, a common measure of how varied a distribution is. We find the blacklisted entropy of China (0.13), Russia (0.23), and Brazil (0.14) are the lowest; Great Britain (0.57), the USA (0.52), and South Africa (0.47) are much higher.
(PNG)

S2 Fig Civic participation against criminal systems free of corruption. Authoritarian regions with low corruption score high, such as the United Arab Emirates (ARE), Singapore (SGP), and Hong Kong (HKG).
(PNG)

S3 Fig Correlation values of actual versus predicted values from the CatBoost regressor, with max depth of 3. (PNG)

S4 Fig SHAP values for e) weighted money-laundering risk score, f) weighted financial transparency score, and g) the financial secrecy index. In analyzing the two controls, we find money laundering risk corresponds to a mixture of blacklist use and offshore diversity, negatively correlated with both civil justice and fundamental rights. Financial transparency resembles more closely offshore diversity, being negatively correlated with order and security but positively correlated with fundamental rights. This means countries with more fundamental rights also use OFCs with more transparency. In other words, while there are overlaps with the weighted scores for money laundering risk and financial transparency, the strategies we are studying are sufficiently distinct from the existing literature.
(PDF)

S1 Table Eight rule of law categories and 44 sub-indicators used in regression models. (DOCX)

S2 Table Full list of sanctioned jurisdictions. (DOCX)
