# Supplementary figures and images for: Secrecy strategies: Global patterns in elites’ quest for confidentiality in offshore finance
Source: PLoS One. 2025 Jul 16;20(7):e0326228. doi: 10.1371/journal.pone.0326228 (PMC12266413; doi:10.1371/journal.pone.0326228)

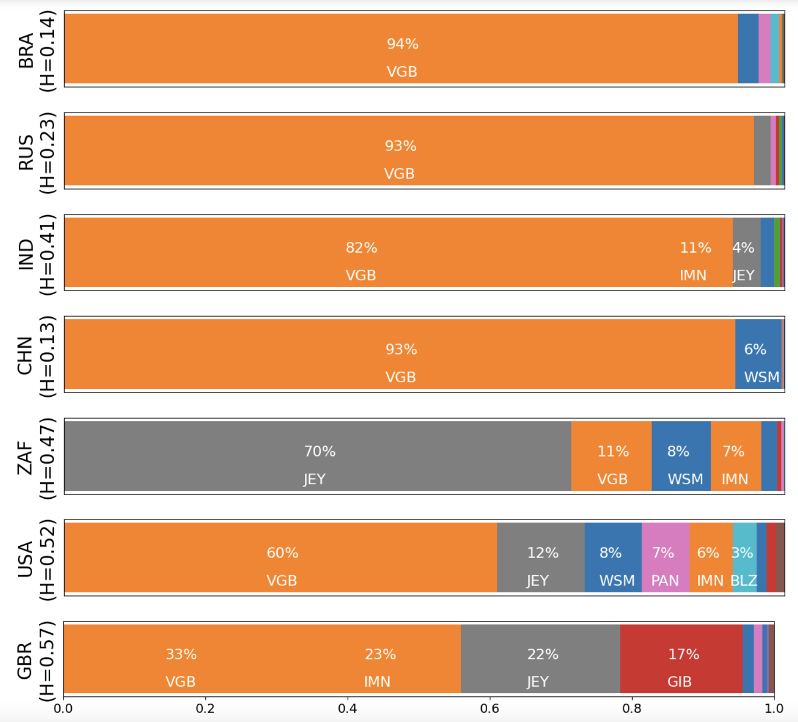

Supplement: S1 Fig — BRICS countries rely dominantly on the British Virgin Islands (VGB). S1 Fig shows the exact distribution of where clients hold their assets in blacklisted regions. Brazil, Russia, India, and China rely almost exclusively on the British Virgin Islands. In contrast, South African clients tend to rely on the Jersey islands. We also quantify the diversity using Shannon Entropy, a common measure of how varied a distribution is. We find the blacklisted entropy of China (0.13), Russia (0.23), and Brazil (0.14) are the lowest; Great Britain (0.57), the USA (0.52), and South Africa (0.47) are much higher. (PNG) [file pone.0326228.s001.png]

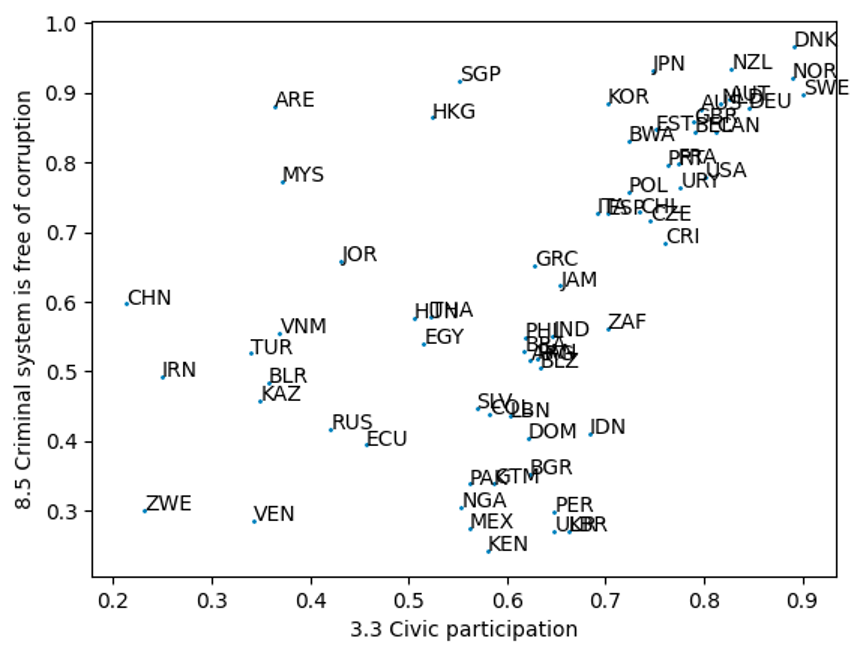

Supplement: S2 Fig — Authoritarian regions with low corruption score high, such as the United Arab Emirates (ARE), Singapore (SGP), and Hong Kong (HKG). (PNG) [file pone.0326228.s002.png]

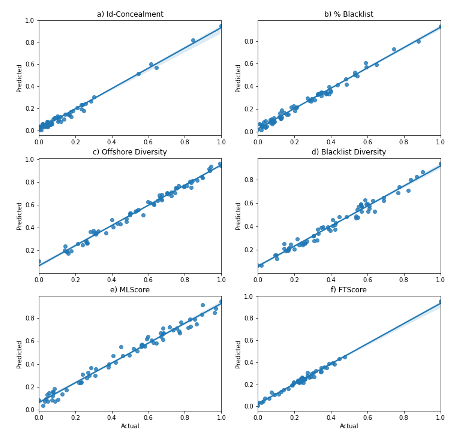

Supplement: S3 Fig — (PNG) [file pone.0326228.s003.png]

e) MLScore

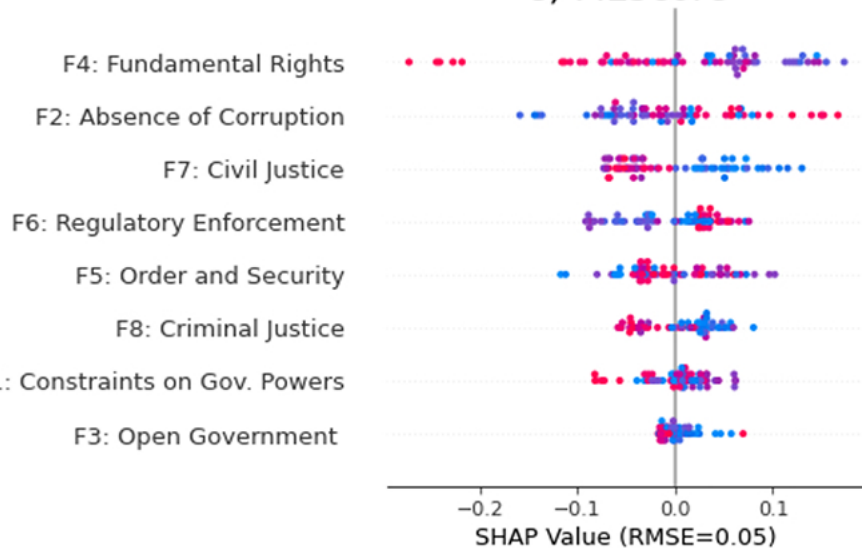

f) FTScore

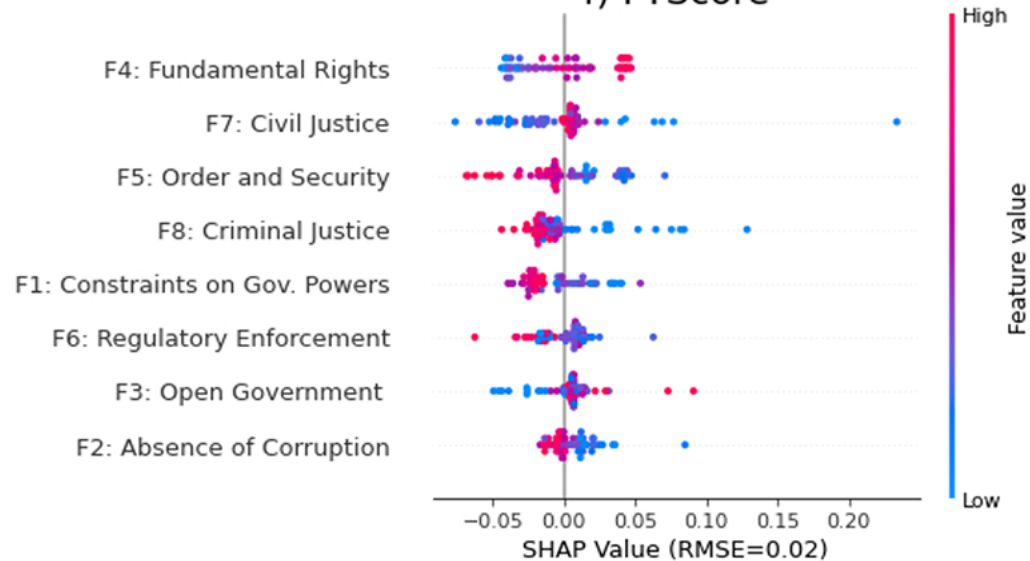

g) FSI

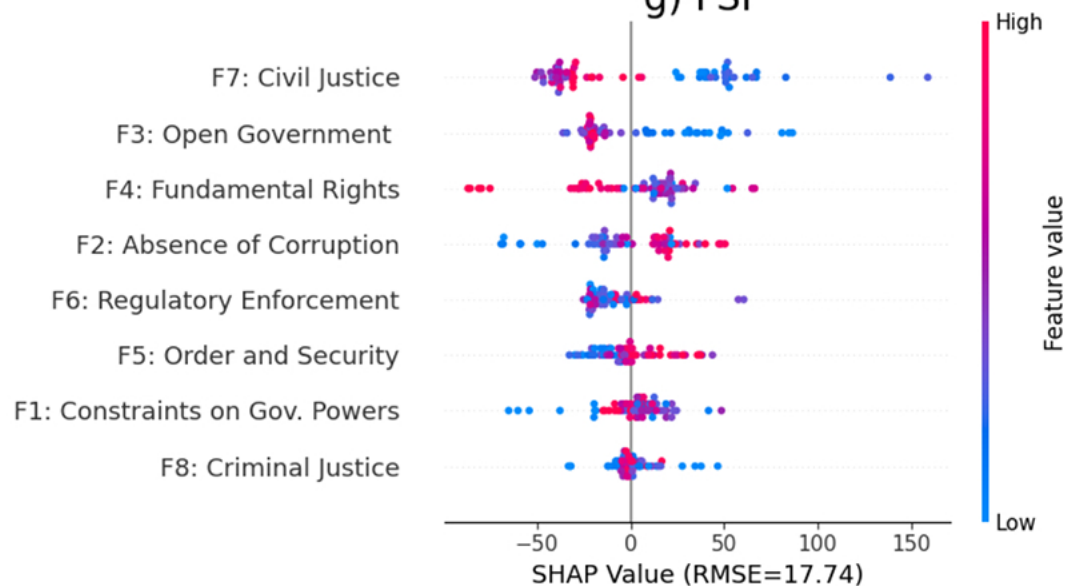

Supplement: S4 Fig — In analyzing the two controls, we find money laundering risk corresponds to a mixture of blacklist use and offshore diversity, negatively correlated with both civil justice and fundamental rights. Financial transparency resembles more closely offshore diversity, being negatively correlated with order and security but positively correlated with fundamental rights. This means countries with more fundamental rights also use OFCs with more transparency. In other words, while there are overlaps with the weighted scores for money laundering risk and financial transparency, the strategies we are studying are sufficiently distinct from the existing literature. (PDF) [file pone.0326228.s004.pdf]
