# Supplementary material for: Secrecy strategies: Global patterns in elites’ quest for confidentiality in offshore finance
Source: PLoS One. 2025 Jul 16;20(7):e0326228. doi: 10.1371/journal.pone.0326228 (PMC12266413; doi:10.1371/journal.pone.0326228)
Supplement: S1 Table — (DOCX) [file pone.0326228.s005.docx]

**Table S1:** Eight rule of law categories and 44 sub-indicators used in regression models.

| Rule of Law Category | Descriptions |
| --- | --- |
| Constraints on Government Powers | 1.1 Government powers are effectively limited by the legislature  1.2 Government powers are effectively limited by the judiciary  1.3 Government powers are effectively limited by independent auditing and review  1.4 Government officials are sanctioned for misconduct  1.5 Government powers are subject to non-governmental checks  1.6 Transition of power is subject to the law |
| Absence of Corruption | 2.1 Government officials in the executive branch do not use public office for private gain  2.2 Government officials in the judicial branch do not use public office for private gain.  2.3 Government officials in the police and the military do not use public office for private gain.  2.4 Government officials in the legislative branch do not use public office for private gain. |
| Open Government | 3.1 Publicized laws and government data.  3.2 Right to information.  3.3 Civic participation.  3.4 Complaint mechanism. |
| Fundamental Rights | 4.1 Equal treatment and absence of discrimination  4.2 The right to life and security of the person is effectively guaranteed.  4.3 Due process of the law and rights of the accused  4.4 Freedom of opinion and expression is effectively guaranteed.  4.5 Freedom of belief and religion is effectively guaranteed.  4.6 Freedom from arbitrary interference with privacy is effectively guaranteed.  4.7 Freedom of assembly and association is effectively guaranteed.  4.8 Fundamental labor rights are effectively guaranteed. |
| Order and Security | 5.1 Crime is effectively controlled.  5.2 Civil conflict is effectively limited.  5.3 People do not resort to violence to redress personal grievances. |
| Regulatory Enforcement | 6.1 Government regulations are effectively enforced.  6.2 Government regulations are applied and enforced without improper influence.  6.3 Administrative proceedings are conducted without unreasonable delay.  6.4 Due process is respected in administrative proceedings.  6.5 The government does not expropriate without lawful process and adequate compensation. |
| Civil Justice | 7.1 People can access and afford civil justice.  7.2 Civil justice is free of discrimination.  7.3 Civil justice is free of corruption.  7.4 Civil justice is free of improper government influence.  7.5 Civil justice is not subject to unreasonable delay.  7.6 Civil justice is effectively enforced.  7.7 Alternative dispute resolution mechanisms are accessible, impartial, and effective. |
| Criminal Justice | 8.1 Criminal investigation system is effective.  8.2 Criminal adjudication system is timely and effective.  8.3 Correctional system is effective in reducing criminal behavior.  8.4 Criminal system is impartial.  8.5 Criminal system is free of corruption.  8.6 Criminal system is free of improper government influence.  8.7 Due process of the law and rights of the accused. |
