# Supplementary material for: Secrecy strategies: Global patterns in elites’ quest for confidentiality in offshore finance
Source: PLoS One. 2025 Jul 16;20(7):e0326228. doi: 10.1371/journal.pone.0326228 (PMC12266413; doi:10.1371/journal.pone.0326228)
Supplement: S2 Table — (DOCX) [file pone.0326228.s006.docx]

**Table S2:** Full list of sanctioned jurisdictions

| List Name | Sanctioned Jurisdictions |
| --- | --- |
| FAFT_2016 | "AFG", "BIH", "GUY", "IRQ", "Lao", "MMR", "PNG", "SYR", "UGA", "VUT", "YEM" |
| FAFT_2017 | "BIH", "ETH", "IRQ", "LKA", "SYR", "TTO", "TUN", "VUT", "YEM" |
| FAFT_2020 | "ALB", "BHS", "BRB", "BWA", "KHM", "GHA", "JAM", "MUS", "MMR", "NIC", "Pak", "Pan", "SYR", "UGA", "YEM", "ZWE" |
| EU_2017 | "ASM", "BHR", "BRB", "GRD", "GUM", "KOR", "MAC", "MHL", "MNG", "NAM", "PLW", "PAN", "LCA", "WSM", "TTO", "TUN", "ARE" |
| EU_2020 | "ASM", "CYM", "FJI", "GUM", "OMN", "PLW", "PAN", "WSM", "SYC", "TTO", "VIR", "VUT" |
| OECD_2000 | "AND", "AIA", "ATG", "ABW", "BHR", "BRB", "BLZ", "VGB", "COK", "DMA", "GIB", "GRD", "IMN", "JEY", "LBR", "LIE", "MDV", "MHL", "MCO", "MSR", "NRU", "ANT", "NIU", "PAN", "WSM", "SEY", "LCA", "KNA", "VCT", "TON", "VIR", "VUT" |
